# Characterizing Malignant Melanoma Clinically Resembling Seborrheic Keratosis Using Deep Knowledge Transfer

**DOI:** 10.3390/cancers13246300

**Published:** 2021-12-15

**Authors:** Panagiota Spyridonos, George Gaitanis, Aristidis Likas, Ioannis Bassukas

**Affiliations:** 1 Department of Medical Physics, Faculty of Medicine, School of Health Sciences, University of Ioannina, 45110 Ioannina, Greece; 2 Department of Skin and Venereal Diseases, Faculty of Medicine, School of Health Sciences, University of Ioannina, 45110 Ioannina, Greece; ggaitan@uoi.gr; 3 Department of Computer Science & Engineering, School of Engineering, University of Ioannina, 45110 Ioannina, Greece; arly@cs.uoi.gr

**Keywords:** melanoma, seborrheic keratosis, SK-like MM, deep learning, knowledge transfer

## Abstract

**Simple Summary:**

Malignant melanomas (MMs) with aypical clinical presentation constitute a diagnostic pitfall, and false negatives carry the risk of a diagnostic delay and improper disease management. Among the most common, challenging presentation forms of MMs are those that clinically resemble seborrheic keratosis (SK). On the other hand, SK may mimic melanoma, producing ‘false positive overdiagnosis’ and leading to needless excisions. The evolving efficiency of deep learning algorithms in image recognition and the availability of large image databases have accelerated the development of advanced computer-aided systems for melanoma detection. In the present study, we used image data from the International Skin Image Collaboration archive to explore the capacity of deep knowledge transfer in the challenging diagnostic task of the atypical skin tumors of MM and SK.

**Abstract:**

Malignant melanomas resembling seborrheic keratosis (SK-like MMs) are atypical, challenging to diagnose melanoma cases that carry the risk of delayed diagnosis and inadequate treatment. On the other hand, SK may mimic melanoma, producing a ‘false positive’ with unnecessary lesion excisions. The present study proposes a computer-based approach using dermoscopy images for the characterization of SΚ-like MMs. Dermoscopic images were retrieved from the International Skin Imaging Collaboration archive. Exploiting image embeddings from pretrained convolutional network VGG16, we trained a support vector machine (SVM) classification model on a data set of 667 images. SVM optimal hyperparameter selection was carried out using the Bayesian optimization method. The classifier was tested on an independent data set of 311 images with atypical appearance: MMs had an absence of pigmented network and had an existence of milia-like cysts. SK lacked milia-like cysts and had a pigmented network. Atypical MMs were characterized with a sensitivity and specificity of 78.6% and 84.5%, respectively. The advent of deep learning in image recognition has attracted the interest of computer science towards improved skin lesion diagnosis. Open-source, public access archives of skin images empower further the implementation and validation of computer-based systems that might contribute significantly to complex clinical diagnostic problems such as the characterization of SK-like MMs.

## 1. Introduction

Malignant melanomas (MMs) with atypical clinical presentation constitute a diagnostic pitfall, and false negatives carry the risk of a diagnostic delay and improper disease management [1,2]. Among the most common, challenging presentation forms of MMs are those that clinically resemble seborrheic keratosis (seborrheic keratosis-like MMs, SK-like MMs) [3].

SK is one of the most frequently diagnosed benign skin tumors in everyday clinical practice. It is a hallmark of aged, chronically sun-exposed skin of older individuals, with well-characterized, in most cases, diagnostic clinical features. The patients are usually alarmed about the sometimes rapidly growing exophytic lesions; however, in most cases, they can be assured that their growths are benign simply based on the clinical examination and without the need for histologic confirmation. Moreover, in many clinically doubtful cases, an additional dermoscopic assessment of the suspect lesion enables a clear-cut diagnosis of the condition based on a series of well-elaborated, typical dermoscopic features [4]. However, none of the SK dermoscopic findings is specific to SK [4], as they can be observed in other skin tumors, including malignant ones, among which are also distinct MM cases (SK-like MMs) [5].

The true incidence of SK-like MM is largely unknown since many of these lesions are misdiagnosed as SK on the basis of the clinical and dermoscopic examination and are not biopsied at this stage [3]. Izikson et al. [6], in a retrospective study covering ten years (1992 to 2001), retrieved 9204 pathology reports of material admitted with the clinical differential diagnosis SK. Melanoma was confirmed by histological examination in 61 of these cases (0.66%).

SK-like melanoma shares clinical and dermoscopic features of SK and melanoma, making the diagnosis challenging. A somewhat regular shape and the presence of benign dermoscopic patterns suggestive of an SK lead to underestimating the true malignant nature of this type of lesion. This ambiguity in the diagnosis was highlighted in a study by Carrera et al. [7] in which 54 dermatologists with about ten years of clinical practice clinically misdiagnosed 40% of 134 SK-like melanomas as benign lesions. An additional dermoscopic evaluation could improve the overall diagnostic accuracy from 60.9 to 68.1%, i.e., not more than by about 20%. Additionally, in the largest dermoscopic study of SK-like melanomas to date, the dermoscopy score and the seven-point checklist score showed benignity range with values 4.2 and 2 [5]. In the same study, Carrera et al. found that the most helpful criteria in correctly diagnosing SK-like MMs, despite the presence of other SK features, were the identification of blue–white veil, streaks, and a pigmented network [5]. Noninvasive optical methods, such as reflectance confocal microscopy (RCM) and optical coherence tomography can be employed to improve accuracy in melanoma diagnosis [8,9,10]. However, in SK-like MMs, the application has been limited due to frequent clinical, dermoscopic misdiagnosis [3].

The diagnostic grey zone between SK and MM becomes even broader as SK mimicking melanomas (MMs-like SK) have also been reported, with an increased risk of false MM diagnoses [11,12,13,14]. Dermoscopy of typical SK is characterized by milia-like cysts, comedo-like openings, and brain-like and finger-like structures [4]. However, pigmented SK can sometimes present dermoscopic patterns that mimic melanocytic lesions, the most frequent of which is the so-called false pigmented network. Dermoscopic evaluation of 402 lesions indicated that pigmented SK could show at least one of the criteria most predictive of melanocytic proliferations [11].

Recent studies have highlighted the contribution of RCM in characterizing MM-like SK. Farnetani et al. [15] retrospectively evaluated RCM images of atypical SK lesions suspicious of MM at dermoscopy to identify a diagnostic approach able to minimize surgical biopsies or excisions. They assessed 111 facial lesions with histological SK diagnosis. By dermoscopy, most lesions (*n* = 83 lesions, 75%) were classified as melanocytic-like. With RCM, only 16% were classified as suspicious of malignancy, with the remaining 84% considered ‘SK-like’. The presence of RCM features associated with typical SK, the rare presence of melanoma-associated features, and the absence of medusa head-like structures seem to be the most sensitive indicators for atypical SK facial lesions.

In another retrospective study, Pezzine et al. [16], applied RCM to analyze excised skin lesions with a ≥1 score of the revisited seven-point dermoscopy checklist [17]. Their objective was to evaluate the agreement of RCM classification and histological diagnoses and the reliability of well-known RCM criteria for SK in identifying SK with atypical dermoscopy presentation. An excellent agreement (97%) was confirmed for RCM and histopathologic examination for SK with atypical dermoscopy presentation, allowing an effective noninvasive differential diagnosis. More importantly, RCM features in this group of atypical lesions were similar to those described for typical SK cases.

Recently, computer-aided diagnosis (CAD) systems are increasingly combined with various noninvasive imaging techniques to encompass advanced image processing and enable the application of artificial intelligence (AI) methods to improve diagnostic accuracy [18,19,20]. In the field of quantitative noninvasive optical techniques, Bozsànyi et al. [21] assessed the usefulness of spectral reflectance and autofluorescence measurements of MM and SK for their accurate differentiation. Using image analysis, they have extracted quantitative autofluorescence intensity measures and created a multiparameter descriptor—the SK index. High values of SK index (resulting from high fluorescence intensity values and the number of highly autofluorescent particles detected in the lesion area) were associated with SK lesions and were mainly caused by the milia-like cysts and comedo-like opening, which are primarily filled with keratin. On the other hand, compared with SK, the melanomas exhibited significantly lower intensity values. The authors used a threshold value of SK index and discriminated SK (*n* = 319) from MM (*n* = 161) with a sensitivity of 91.9% and specificity of 57.0%. It is worth noting that their data set included six image sets of MM-like SK and 52 image sets of SK-like MM; however, they did not clarify the clinical or dermoscopic atypia criteria of these latter cases.

In the same context, Wang et al. [22] developed a support vector machine (SVM) classification model fed with speckle patterns estimated from image histogram of copolarized and cross-polarized speckle images and a depolarization ratio image D to differentiate between MM and SK. Using a data set of 143 patients (MM *n* = 37, SK *n* = 106), they could discriminate SK from MM with this approach with a sensitivity of 87.63% and a specificity of 85.74%.

The increasing worldwide integration of dermoscopy in clinical dermatology practice [23,24], the evolving efficiency of deep learning algorithms in image recognition, and the availability of extensive image archives have greatly accelerated the development of advanced CAD systems for melanoma detection [25,26,27,28,29,30]. Earlier efforts were mainly concentrated on discriminating benign melanocytic lesions from MM. However, with the availability of large image datasets, the interest has shifted towards a more sophisticated categorization of skin tumors. Today, the largest, publicly available dataset of dermoscopic images is the International Skin Image Collaboration (ISIC) archive [31]. ISIC promotes CAD-based research by sponsoring annual related challenges for the computer science community in association with leading computer vision conferences. Thus in recognition of the immense clinical impact of differentiating between MM and SK, in 2017 ISIC released a focused dataset with a three-task challenge: lesion segmentation, visual dermoscopic features detection, and lesion discrimination firstly between melanoma vs. nevus and seborrheic keratosis (malignant vs. benign lesions), and secondly between seborrheic keratosis vs. nevus and melanoma (nonmelanocytic vs. melanocytic lesions) [32].

In the present study, we used image data from the ISIC archive to investigate the discrimination efficiency of image embeddings derived from pretrained convolutional network VGG16 to differentiate between MM and SK in the challenging diagnostic task of the preinvasive diagnosis of SK-like MMs. To the best of our knowledge, this study is the first effort exploring the capacity of deep knowledge transfer in refined complexity diagnostic tasks of clinically atypical skin tumors.

## 2. Materials and Methods

### 2.1. Data Set Description

Our data set comprised 978 dermoscopic images (malignant melanoma, MM, *n* = 550; seborrheic keratosis, SK, *n* = 428) retrieved from the International Skin Image Collaboration archive [31]. Patients’ metadata are summarized in Table 1.

The clinical diagnosis of all MM cases and of 310 SK cases (72.4%) was confirmed by histological examination.

A large part of the images came from ISIC 2017 challenge [32]. This database provides ground truth lesion images with annotation of the lesion area and the dermoscopic patterns. To enhance our training set, we retrieved 200 additional images (*n* = 100 MM, *n* = 100 SK; the BCN_20000 dataset, Hospital Clínic de Barcelona) from the ISIC archive. For the remaining images (BCN_2000 dataset), the lesion area was annotated manually by our experts. The study did not include images in which hair (or another type of noise such as bubbles) substantially corrupted the lesion area. The image resolution in the dataset ranged from 639 × 602 to 6720 × 4461 pixels.

**Figure 1:**
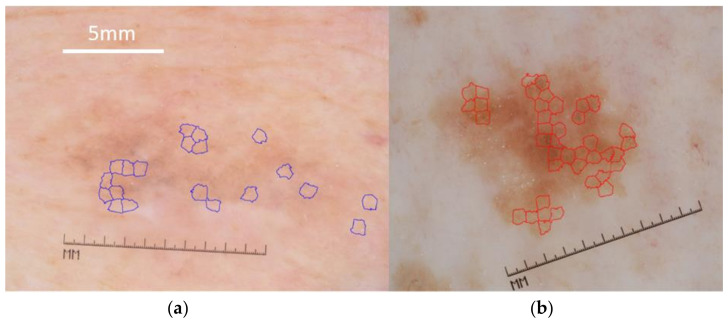
Atypical cases: (**a**) MM with milia-like cysts (annotated) (**b**) SK with a pigmented network (annotated). Scale bar = 5mm applies to both panels. Images in the figure were adapted to a uniform magnification (compare same lengths of the original integrated dermatoscope scale) Figures are available online [31].

**Figure 2:**
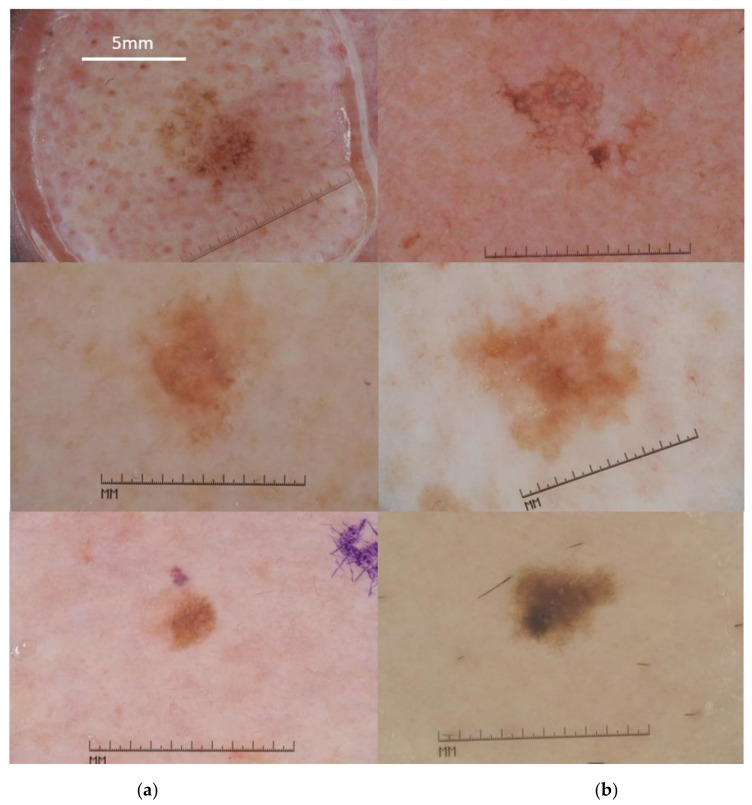
Examples of MM (**a**) and SK (**b**) cases. Pairs (left-right) of selected cases are displayed to highlight the distinct overlap of the morphological features. Scale bar = 5mm applies to all panels. All images in the figure were adapted to a uniform magnification (compare same lengths of the original integrated dermatoscope scale) (Figures are available online [31]).

To train our system, we used *n* = 349 cases of MM and *n* = 318 cases of SK. The inclusion criteria of dermoscopic images in the test set (MM *n* = 201, SK *n* = 110) were the presence of at least one atypical dermoscopy pattern. For MM, this is the absence of pigmented network or the presence of milia-like cysts (or both). On the other hand, atypical SK lacked milia-like cysts or had a pigmented network (or both) (Figure 1 and Figure 2).

### 2.2. Feature Extraction Using Deep Knowledge Transfer

The objective of machine learning in CAD systems is to extract patterns from images and use these patterns to make diagnostic predictions. These patterns are feature vector representations of input images, also called embeddings. From the deep learning perspective, using pretrained embeddings to encode images into feature vectors is known as transfer learning [33]. A typical example is to repurpose pretrained embeddings trained on a large corpus of millions of images [34] for a large-scale classification task to implement a classification model for a different classification task, with much fewer data available.

Several studies have indicated that embeddings extracted from deep convolutional neural networks (CNNs) are powerful for various visual recognition tasks [35,36,37]. Their outstanding performance as image representation learners grew the trend of utilizing them as optimized feature generators for skin lesion classification [38,39,40,41,42,43]. Our work, aligned with previous research evidence, explores the efficiency of the pretrained CNN, namely the VGG16 [44] as the starting point, for the generation of image embeddings in order to discriminate between cases of atypical MM and atypical SK.

**Figure 3:**
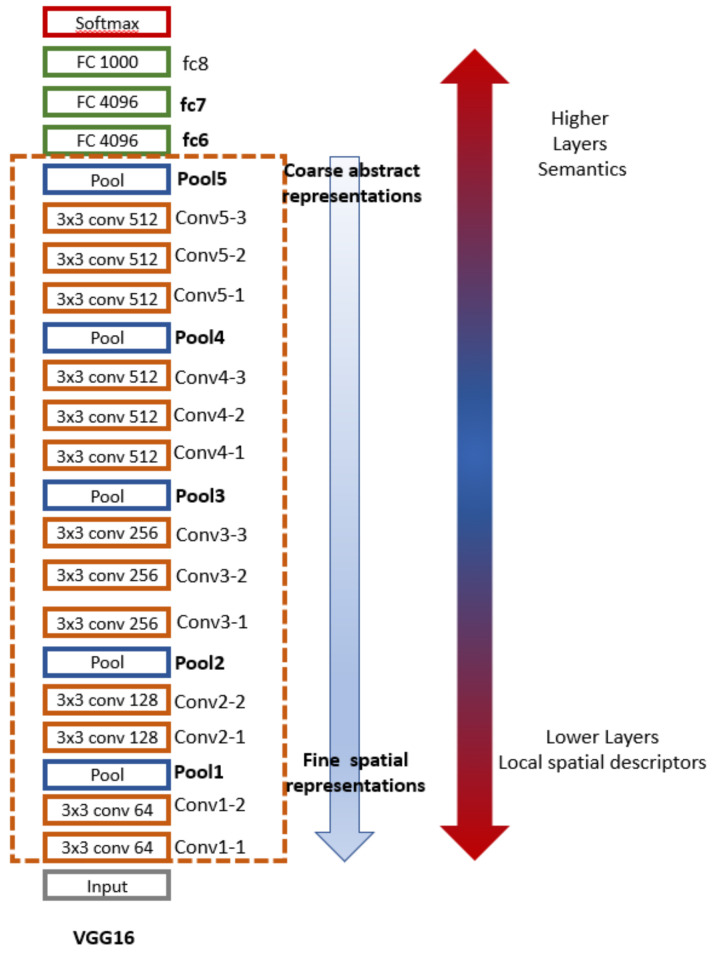
VGG16 architecture and the image representation hierarchies.

As a conventional deep CNN, VGG16 is a 16-layer architecture that consists of convolutional and fully connected parts. VGG16 pretrained on ImageNet is a classifier architecture for distinguishing a large number of object classes [34]. This goal is achieved gradually by learning image representations in a hierarchical order (Figure 3).

Top layers capture more abstract and high-level semantic features. They are robust at distinguishing objects of different classes (i.e., flowers, dogs, etc.) even at significant appearance changes or in the presence of a noisy background. Still, they are less discriminative to objects of the same category (i.e., differentiate between different species of flowers). Moreover, several studies confirmed that the fully connected layers of the CNN, whose role is primarily that of classification, tend to exhibit relatively worse generalization ability and transferability [45]. In contrast, the lower convolutional layers provide more detailed spatial representations. They are more helpful to localize fine-grained details and distinguish a target object from its distracters (other objects with similar appearance, i.e., distinguish between bird species). However, they are less robust to appearance changes. The convolutional layers, acting progressively from fine, spatial to coarse, abstract representations generally transfer well [33,37,45,46] to diverse classification tasks. Based on this evidence, in the present work, we aimed to find the optimal transition point in the convolutional layers to mine high-capacity image representations for the challenging diagnostic task of SK-like MMs characterization.

We exploited image representations from the layers “pool2–pool5”. For comparison purposes, we also extracted the fully connected layers’ “FC6”, “FC7” feature maps so that we can contrast the behavior of the convolutional and fully connected layers (Figure 3).

Finally, the efficiency of VGG16 representations was compared with hierarchical feature embeddings from the ResNet50 convolutional network [47]. Image encoding from fine spatial to coarse abstract, was explored using the layers ReLU_10, ReLU_22, ReLU_40, and ReLU_49.

The image representation of a convolutional layer (activation) forms a tensor of HxWxd, consisting of d feature maps of size H × W. Each feature map is flattened using global average pooling to produce a d-dimensional feature vector. Table 2 summarizes the different VGG16 and ResNet50 layers’ representations and their resulting feature vectors for an input image of 224 × 224 × 3 pixels.

### 2.3. Implementation and Evaluation of the Diagnostic Model

The extracted deep feature vectors (Table 2) were used to train different binary SVM classifiers. SVM is the classifier of choice for assessing representations from pretrained CNNs [35,36]. For all SVM models, optimal hyperparameter selection (Box Constraint, Kernel function, Kernel scale, Polynomial order) was carried out using the Bayesian optimization method [48] that minimizes k-fold (k = 5) cross-validation classifier error. For each model, the accuracy performance was evaluated in an independent data set of challenging cases of MM and SK in terms of sensitivity, specificity, and overall accuracy:

(1) $$Sensitivity=\frac{TP}{TP+FN}$$

(2) $$Specificity=\frac{TN}{TN+FP}$$

(3) $$Accuracy=\frac{TP+TN}{TP+FP+TN+FN}$$

where TN is the number of SK correctly identified, FN is the number of MM incorrectly identified as SK, TP is the number of MM correctly identified, and FP is the number of SK incorrectly identified as MM. 

The models’ accuracies were assessed with the McNemar test to detect whether the misclassification rates between any of the two models were statistically significant or not [49,50].

### 2.4. Image Preprocessing

**Figure 4:**
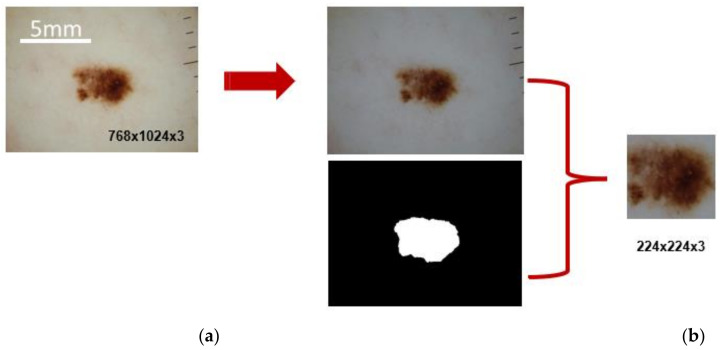
Image preprocessing example. (**a**) Each image is color normalized and combined with the lesion image mask to produce (**b**) the final lesion-cropped and adequately resized input to the CNN model. Scale bar = 5mm. (Figure available online [31]).

Before being used as input to pretrained CNNs, all images were preprocessed following a standard pipeline of color normalization, cropping, and resizing (Figure 4). To achieve a color constancy in the whole data set, we used the Grey world color constancy method [51], initially used by [52] and followed by many researchers in automated skin classification works [53,54,55]. Finally, the exact lesion dimensions were used to crop the images as proposed in [55].

## 3. Results

Bayesian optimization was run for 100 iterations, and different image embeddings from pretrained VGG16 and ResNet50 layers resulted in different classification models, with noticeable differences in test classification accuracies (Table 3).

The SVM model with a gaussian kernel using feature vectors from the ‘pool3’ layer exhibited the best overall accuracy of 80.7% (251/311 cases) and a sensitivity and specificity of 78.6% (158/201 cases) and 84.5% (93/110 cases), respectively. The highest specificity, 90.9% (100/110 cases), was achieved by a linear SVM classifier and features from the convolutional layer ‘pool4’. Considering the ResNet50 approach, there was also the SVM model with a gaussian kernel using feature vectors from the ‘ReLU_22’ layer that exhibited the best overall accuracy of 79.4% with a sensitivity and specificity of 76.1% and 85.4%, respectively.

More detailed comparison results are illustrated in Table 4, where the statistical significance (McNemar test) of the differences in the observed accuracies is displayed. Considering the VGG16 embeddings, layer ‘pool3’ produced significantly better sensitivity and overall accuracy with more than a 99.9% confidence level. The ‘pool4’ layer outperformed the sensitivity and overall accuracy of pool5 and FC7 layers with a confidence of more than 95% and those of layers pool2 and FC6 with a confidence of more than 99.9%. The fully connected layer FC7 outperformed the FC6 layer in sensitivity with more than 95% confidence.

Overall, all the representations resulted in comparable levels of specificity.

## 4. Discussion

The importance of the timely diagnosis of difficult to recognize melanomas that can clinically resemble benign tumors, such as the SK-like MMs, has been emphasized in previous studies [3,5,7,55,56]. Carrera et al. have indicated specific dermoscopic criteria for correctly identifying such challenging SK-like MM cases [5]. On the other hand, given their larger numbers and significant dermoscopic variability, SK may, at times, mimic melanoma contributing to the clinical MM overdiagnosis [14,15]. RCM may help diagnose challenging cases [3], and recent studies have highlighted the ability of RCM patterns to identify SK with atypical dermoscopy presentation [15,16]. However, there is a lack of related RCM studies focusing on SK-like MM [3]. Moreover, these later studies [5,15,16] have unilaterally highlighted the diagnostic accuracy of dermoscopic and RCM features. The dermoscopic features that assist experts in characterizing SK-like MM have not been employed to assess atypical cases of SK, and the specific RCM patterns were not evaluated in SK-like MM cases. Moreover, the use of RCM is time-consuming, and the increased cost of the equipment restricts the wide availability of this technology.

Today, with the rapid advancement of deep learning methods and the publicly available data sets, dermoscopic images almost monopolize the research interest of CAD skin lesion systems. Numerous breakthrough studies, mainly from the field of computer science, have demonstrated high (expert-level) accuracy in melanoma detection. These high accuracy rates are either related to binary classification tasks as benign vs. malignant or multidifferential diagnosis tasks. In this study, we explored the potential of deep knowledge transfer to approach the challenging ‘grey zone’ of atypical cases of MM and SK. Studying the different image representation transfer results from a well-known VGG16 architecture and following a standard workflow, we achieved a sensitivity of 78.6% and a specificity of 84.5% using the convolutional layer ‘pool3’ as a feature extractor. Our results confirm that meaningful feature reuse is concentrated at the convolutional layers rather than at higher, fully connected layers [33,36]. We have also tested the ResNet50 network, and we have verified the existence of the optimal transition from fine spatial to coarse semantic features through the deeper convolutional blocks of ResNet. However, since the discriminating image embeddings are located at the middle layers, the middle-level image embeddings from ResNet50 are of comparable capacity to that of middle-level VGG16.

Moreover, a meta-analysis of 70 studies on CAD systems, published between 2002 and 2018 [19], gave a melanoma sensitivity of 0.74 (95% CI, 0.66–0.80) and a specificity of 0.84 (95% CI, 0.79–0.88), indicating that we have tackled the challenging discrimination of SK-like MMs with comparable accuracies.

In future work, aggregating methods to combine embeddings from middle convolutional layers of the same network or different networks in a global, dense image representation might further boost the system’s accuracy. However, the availability of annotated and high-quality image data remains the key contributor to improving accuracy.

Our present contribution is thus twofold: Firstly, the comprehensive evaluation of the transferability of features from different layers of pretrained VGG16 and ResNet50 unveiled the excellent efficiency and generalization properties of the middle-level convolutional layers. Secondly, we targeted a challenging diagnostic task where key dermoscopic patterns of either condition are shared between benign and malignant lesions. It is worth noting that the herein proposed CAD system is aligned with the recent technological advances in smartphone-based teledermatology that promise to enhance diagnostic efficacy at the clinical level [57].

The main limitation of this study is that the feature extraction from pretrained image embeddings is acting more like a black box. The exploited image patterns generate little human interpretable evidence of lesion diagnosis. The effectiveness of this algorithm in prediagnosed cases is within the scopes of a future prospective study.

## 5. Conclusions

Deep learning has boosted the efficiency of CAD systems significantly. With the publicly available data collections, the computer science community has now the opportunity to test the accuracy of these systems in melanoma diagnosis. Moreover, when these systems clearly focus on a specific diagnostic task and are trained and tested sufficiently, they may support dermatologists in challenging diagnostic tasks.

## Figures and Tables

**Table 1:** Patient metadata: Gender and age of the patients.

Patient Characteristics	MM	SK
Female	240	195
Male	248	230
Undefined	62	3
Mean Age	60.8	64
Median Age	65	65
Standard Deviation (SD) of Age	15.9	13.3

MM: malignant melanoma, SK: seborrheic keratosis.

**Table 2:** VGG16 and ResNet50 pretrained image representations and their corresponding d-dimensions feature vectors by global averaging. CNN: convolutional neural network.

CNN	Layer	Imager Representation (Activation)	Feature Vector Dimension (d)
VGG16	Pool2	56 × 56 × 128	128
	Pool3	28 × 28 × 256	256
	Pool4	14 × 14 × 512	512
	Pool5	7 × 7 × 512	512
	FC6	1 × 1 × 4096	4096
	FC7	1 × 1 × 4096	4096
ResNet50	ReLU_10	56 × 56 × 256	256
	ReLU_22	28 × 28 × 512	512
	ReLU_40	14 × 14 × 1024	1024
	ReLU_49	7 × 7 × 2048	2048

**Table 3:** SVM classification models performance using different image representations. Bold annotation highlights the best performance yielded by VGG16 and ResNet50, respectively.

CNN	Layer	SVM Model	Sensitivity (%)	Specificity (%)	Accuracy (%)
VGG16	Pool2	Polynomial	56.7	86.4	67.2
	**Pool3**	**Gaussian**	**78.6**	**84.5**	**80.7**
	Pool4	Linear	68.2	90.9	75.2
	Poo5		59.2	85.4	68.5
	FC6		57.2	86.4	67.5
	FC7		62.2	82.7	69.4
ResNet50	ReLU_10	Polynomial	68.1	86.4	74.6
	**ReLU_22**	**Gaussian**	**76.1**	**85.4**	**79.4**
	ReLU_40	Linear	70.6	89.1	77.2
	ReLU_49		62.7	86.4	71.1

**Table 4:** Cross-comparison of the classifiers’ accuracies (McNemar test). The arrowheads point to the classifier with the highest accuracy, and the lines denote comparable accuracies. The overall accuracy, sensitivity, and specificity results are denoted with dark, red, and blue colors. For example, comparing the performance of layers’ representations FC6 and FC7, the FC7 layer exhibited statistically higher sensitivity with a confidence level of more than 95%. Only *p*-values of significantly different outcomes are displayed.

	Pool2		Pool4		Pool5		FC6		FC7		ReLU_22	
**Pool3**	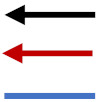	*p* < 0.001	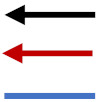	*p* < 0.001	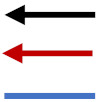	*p* < 0.001	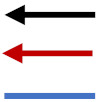	*p* < 0.001	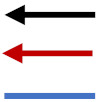	*p* < 0.001	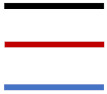	
**Pool2**	-		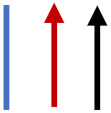	*p* < 0.001	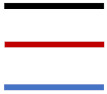		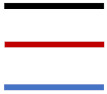		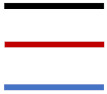		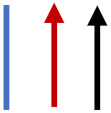	*p* < 0.001
**Pool4**	-		-		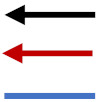	*p* < 0.05	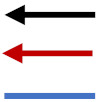	*p* < 0.001	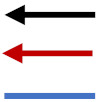	*p* < 0.05	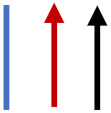	*p* < 0.001
**Pool5**	-		-		-		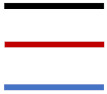		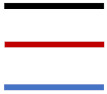		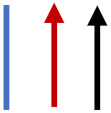	*p* < 0.001
**FC6**	-		-		-		-		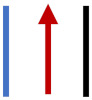	*p* < 0.05	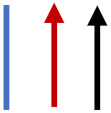	*p* < 0.001
**FC7**	-		-		-		-		-		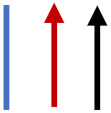	*p* < 0.001

## Data Availability

Publicly available datasets were analyzed in this study. This data can be found here: https://challenge.isic-archive.com/data/ 2017 challenge. Last accessed date 9 December 2021.
